# V1 neurons can distinguish between motion in the world and visual displacements due to eye movements: a microsaccade study

**DOI:** 10.1186/1471-2202-14-S1-P262

**Published:** 2013-07-08

**Authors:** Xoana G Troncoso, Ali Najafian Jazi, Jorge Otero-Millan, Stephen L Macknik, Susana Martinez-Conde

**Affiliations:** 1 Barrow Neurological Institute, Phoenix, Arizona 85013, USA; 2 Unité de Neurosciences Information et Complexité (UNIC), CNRS, Gif sur Yvette, 91198, France; 3 Arizona State University, Tempe, Arizona 85287, USA; 4 University of Vigo, Vigo, 36310, Spain

## 

A major question in neuroscience concerns how perceptual systems differentiate between self-motion and motion in the world. This problem has special importance in vision, where the oculomotor system can track moving objects and move the fovea rapidly to sequential targets of interest, in conditions in which both observer and target are moving. We can easily distinguish between real world motion and a comparable displacement of the image over the retina due to an eye movement, despite equivalent retinal stimulation for externally and internally generated motion. However, how the brain performs this operation or which brain areas are involved remains unknown.

Here we recorded single neuron activity from awake monkey area V1 and compared the responses triggered by microsaccades (small-magnitude saccades that occur while attempting to fixate) to the responses induced by stimulus motion mimicking microsaccades. Our experimental set up allowed us to determine 1) whether area V1 neurons can differentiate between internal and external motion, and 2) the contribution of retinal versus non-retinal sources to microsaccade-driven neuronal responses in area V1. Because microsaccades displace receptive fields over an image, the interplay between receptive field and visual stimulus might fully explain area V1 responses to microsaccades. If so, real microsaccades should elicit the same responses as stimulus motion that mimics microsaccades. Alternatively, responses to real and simulated microsaccades might be different, indicating that responses to real microsaccades include inputs from both retinal and non-retinal sources (such as corollary discharge from the oculomotor system in association with microsaccades, proprioceptive signals from the eye muscles, and/ or global motion integration).

We found that neuronal responses to real microsaccades were generally biphasic: a quick and dramatic increase over baseline was typically followed by a smaller and slower trough below baseline, whereas responses to simulated microsaccades included an excitatory peak but no trough. These findings suggest that excitatory responses to real microsaccades result from the displacement of the visual stimulus over the classical receptive field, with the subsequent inhibition reflecting non-retinal sources. The differential neural response to real versus simulated microsaccades further indicates that area V1 neurons can distinguish between internally and externally generated motion (i.e. visual displacements due to eye movements versus actual motion in the world). These findings help to delineate and constrain the role of area V1 in information processing, visual stability and/or perceptual suppression during microsaccades.

